# Counteracting Static Stretching-Induced Anaerobic Performance Impairment: The Role of Caffeine

**DOI:** 10.5114/jhk/207251

**Published:** 2025-09-23

**Authors:** Refik Çabuk, Onur Demirarar, Hakan Arslan, Merve Cin, Bahtiyar Özçaldıran, Bettina Karsten

**Affiliations:** 1 Department of Coaching Education, Yaşar Doğu Faculty of Sport Sciences, Ondokuz Mayıs University, Samsun, Türkiye.; 2 Department of Physical Education and Sports Sciences, Gendarmerie and Coast Guard Academy, Ankara, Türkiye.; 3 Department of Sports and Health Sciences, Institution of Health Sciences, Ege University, Izmir, Türkiye.; 4 Department of Coaching Education, Faculty of Sports Sciences, Ege University, Izmir, Türkiye.; 5 Faculty of Health, Pedagogy & Social Science, CBS University of Applied Science, Cologne, Germany.; 6 Institute for Lifecourse Development, School of Human Sciences, Centre for Exercise Activity and Rehabilitation, University of Greenwich, London, United Kingdom.

**Keywords:** anaerobic power, anaerobic capacity, neural inhibition, stretching, Wingate

## Abstract

Static stretching (SS) practices can result in acute anaerobic performance reductions with an associated reduction in neural muscle input. The purpose of this study was to determine whether the neural stimulus of caffeine intake would sufficiently minimize or remove the potential inhibitory effect of acute SS on anaerobic performance measured by a 30-s all-out Wingate Anaerobic Test (WAnT). Twelve (12) recreational male athletes performed the WAnT under six conditions, namely a no-treatment control condition, an SS condition (nine lower-body SS exercises), a placebo condition (6 mg∙kg^-1^ maltodextrin), a placebo combined with SS condition, a caffeine condition (6 mg∙kg^-1^) and a caffeine combined with SS condition. Peak power output (PPO), average power output (AvPO) and maximal revolutions per minute (RPM_max_) were measured. SS resulted in significantly lower PPO values (p = 0.005), RPM_max_ values (p = 0.014), and longer tPPO (p = 0.036) compared to the control condition. The condition of SS in combination with caffeine intake resulted in significantly higher PPO (p = 0.004), AvPO (p = 0.025) and RPM_max_ (p = 0.000) values compared to the condition of SS only. In addition, the control condition showed significantly lower values in PPO (p = 0.029), AvPO (p = 0.008), and RPM_max_ (p = 0.018) variables compared to the caffeine condition, whereas no significant difference (p = 0.260–0.567) was observed when compared with the caffeine and SS combination condition. The results of this study confirm the negative effects of SS on anaerobic performance while demonstrating that caffeine intake may minimize or counterbalance these effects. Additionally, the potential risk that SS may partially diminish the positive effects of caffeine should not be overlooked.

## Introduction

One integral part of a warm-up routine comprises stretching. In this respect, following light or moderate aerobic exercise, athletes often warm up with bouts of acute static stretching (SS) exercises (Behm and Chaouachi, 2011). Research has demonstrated that SS increases musculoskeletal flexibility by affecting both mechanical (Cè et al., 2015) and neurological (Behm et al., 2021) properties of the musculoskeletal unit. Stretching warm-up routines can also enhance athletic performance by increasing body temperature, accelerating action potential conduction, and increased metabolic activity (Chaabene et al., 2019). Although the benefits of stretching on athletic performance are well known, the use of longer duration SS (i.e., ≥ 60 s) exercise before competition remains controversial (Chaabene et al., 2019). Research examining the effects of SS on speed and power production has typically revealed impairment after holding stretches for ≥ 60 s, while shorter duration of SS (i.e., ≤ 60 s) appears to demonstrate a less negative impact (Behm et al., 2016). Studies have compared the Wingate test (WAnT) performance following SS with WAnT performance after a standard cycling warm-up (Miller, 2020; Velasque et al., 2020). Velasque et al. (2020) compared the WAnT performance indices after SS exercises (2 x 2 x 45 s, rest: 60 s) with those obtained after a standard cycling warm-up protocol in male soccer players with low (LP) and high performance (HP) based on Yo-Yo Intermittent Recovery level 2 test results. Their results revealed that the SS protocol led to lower peak and average power output (PPO and AvPO, respectively) in the LP group, whereas no decrease was observed in the HP group. In another study, Miller (2020) observed that in recreationally active male participants, PPO values in the WAnT were lower after the SS protocol (4 sets × 4 stretching exercises × 30 s, rest: 30 s) compared to the traditional cycling warm-up protocol, while AvPO and minimum power output (POmin) values remained similar. In the study conducted by Franco et al. (2012) with recreational male participants, no significant difference was found in AvPO and PPO after the SS protocol (3 × 3 SS exercises × 30 s), whereas a decrease in POmin and an increase in time to reach PPO (tPPO) were detected. These results indicate that SS can acutely reduce anaerobic performance and that the WAnT can accurately detect these changes. The differences in results across those studies can be explained by factors such as the fitness level, the number of repetitions, the duration of each repetition, the muscle groups stretched, and the total stretching time (Franco et al., 2008; Velasque et al., 2020).

The consensus in the literature indicates that SS of long duration tends to have negative effects, particularly on activities requiring anaerobic performance (Behm et al., 2016). The most widely accepted explanation for performance declines due to long SS practices, in the context of peripheral mechanisms, refers to changes in the viscoelastic properties of the muscle-tendon unit (Cè et al., 2015; Konrad et al., 2019). These changes increase the flexibility of the muscle-tendon unit while reducing its stiffness (Behm and Chaouachi, 2011; Kallerud and Gleeson, 2013; McHugh and Cosgrave, 2010). Increased muscle-tendon unit flexibility may reduce the amount of elastic energy stored during the stretching phase of the stretch-shortening cycle (SSC) activities. Additionally, greater muscle-tendon unit compliance may alter the muscle’s length-tension relationship, leading to a decrease in force production capacity (Kallerud and Gleeson, 2013). This can result in performance losses in SSC-based movements (Kallerud and Gleeson, 2013). Although the exact mechanisms behind SS-induced impairment in athletic performance have not yet been fully clarified (Behm et al., 2021), several studies (Trajano et al., 2013, 2017, 2021) have shown that the decrease in anaerobic performance following prolonged SS is largely related to reduced central nervous system (CNS) stimulation or a decline in the efferent neural drive. To prevent such performance impairment, Kurt et al. (2023, 2024) recommended using dynamic stretching instead of SS before anaerobic exercises. When properly applied, dynamic stretching protocols have a range of positive effects on anaerobic performance (Kurt et al., 2023), including increased muscle-tendon unit stiffness, neuromuscular activation, blood flow, and body temperature (Behm and Chaouachi, 2011; Hough et al., 2009). However, SS is still widely preferred by coaches and athletes because it is more effective in increasing flexibility, joint range of motion, and reducing the incidence of injuries related to physical activity (Chaabene et al., 2019; Kamandulis et al., 2024). On the other hand, the tradition of including SS as part of warm-up routines has persisted since the 20th century (Behm, 2018) and remains a common practice, especially in certain sports and among traditional coaches. Additionally, giving up SS before exercise may have negative psychological effects on an athlete who has this habit or believes it is necessary (Young, 2007). This belief and habitual practice contribute to the continued use of SS, regardless of scientific debates (Popp et al., 2017; Young, 2007). Behm et al. (2016) calculated a small overall performance loss of 1.3% in power- and speed-related activities following the SS protocol. They also stated that this decline was insufficient to justify the complete removal of SS from warm-up protocols.

On the other hand, in certain sports (e.g., sprinting, the long jump, the high jump, the shot put, the javelin throw, etc.), these reductions may be practically significant. Moreover, for elite athletes, a mere 1% decrease in performance could result in failing to qualify for the Olympics or missing out on a medal. Therefore, there is a need to develop effective strategies to eliminate or minimize performance losses that may arise due to prolonged SS. As stated by Behm and colleagues (2016), a large portion of existing research suggests that the most likely cause of performance declines following SS is the reduction in the CNS drive. In this regard, if inhibition of neural input is considered the primary source of this mechanism, caffeine, as an external neural stimulant, may offer a potential solution to minimize or completely eliminate the expected performance losses. One of the main performance-enhancing effects of caffeine is its stimulating role in the CNS (Davis and Green, 2009). Caffeine acts as an adenosine receptor antagonist, blocking the inhibitory effects of adenosine in the CNS (Davis and Green, 2009). As a result, neuronal activity increases, and alertness along with concentration improve (Davis and Green, 2009). The blockade of adenosine leads to an increase in the release of neurotransmitters such as dopamine and noradrenaline, facilitating voluntary muscle activation (de Kivit et al., 2013; Fredholm et al., 1999). As a result, caffeine is expected to create a higher drive at the CNS level and reduce neuromuscular inhibition caused by SS. Indeed, it has been suggested that the performance loss observed after SS results from increased neuromuscular inhibition originating from the CNS and that the use of a stimulant such as caffeine may reduce this inhibitory effect (Behm et al., 2016). This strategy may help athletes benefit from the advantages of pre-activity SS while avoiding its negative effects.

However, there is a limited number of studies on this topic. To our knowledge, only one study by Farney et al. (2019) has proposed the hypothesis that caffeine may counteract performance decline induced by SS by stimulating the CNS and increasing motor unit activation. In their study, a one-repetition maximum (1RM) knee flexion test was used, and the researchers administered a protocol consisting of a 6 mg∙kg^-1^ caffeine dose and 4 × 4 SS exercises (each lasting 30 s). Their results indicated that caffeine intake did not reduce the loss in 1RM knee flexion strength observed after the SS protocol. Due to the lack of proper control conditions, it was not possible to clearly distinguish between the effects of caffeine and placebo conditions. Additionally, some review studies have stated that caffeine intake enhances muscular endurance performance but has no effect on maximal strength (Davis and Green, 2009; Polito et al., 2016) or that its effects remain inconclusive (Wilk et al., 2019). Therefore, in the study by Farney et al. (2019), caffeine, which had already been shown to have no effect on 1RM knee flexion test results, may have failed to counteract or mitigate the negative effects of SS. More research is needed to explore different performance measures. It remains unclear how caffeine intake after SS affects anaerobic power and capacity. Therefore, our study aimed to determine whether caffeine intake would sufficiently minimize or completely eliminate the potential inhibitory effect of acute SS on WAnT performance indices. Based on the literature, the following hypotheses were formulated: (i) 60-s SS would negatively affect anaerobic performance, and (ii) caffeine intake combined with 60-s SS would counteract the SS-induced impairment in anaerobic performance.

## Methods

### Participants

A total of 12 recreational male athletes (age: 22.2 ± 2.4 years; body height: 179 ± 4.6 cm; body mass: 70.7 ± 9.7 kg; body mass index; 22.1 ± 2.1 kg^.^m^-2^; body fat percentage; 8 ± 5.4%) completed the study. All participants were sports science students of the local university. All participants had a background in competitive sports during their adolescence or high school years; however, in recent years, they had shifted toward recreational physical activity. At the time of the study, they participated in strength training, team sports, moderate- to high-intensity running, and other types of exercise at least three days per week, and had a minimum of two years of experience in recreational training. None of the participants were involved in professional or elite-level sports.

The study was approved by the Ege University Clinical Research Ethics Committee (protocol code: 21-6.1T/44; approval date: 24 June 2021) and conducted in accordance with the Declaration of Helsinki. Prior to providing written informed consent, participants were fully informed of the nature and potential risks of the study. Participants who consumed more than 60 mg of caffeine per day and those who had an allergic reaction to caffeine were excluded from the study. Participants were required to refrain from alcohol, caffeine supplements, or other ergogenic substances during the testing period. Additionally, all participants were informed about products containing caffeine. To minimize the potential effect of reduced caloric intake on exercise performance, participants were requested to consume their typical diet (including zero caffeine consumption). During each visit to the laboratory, participants were questioned whether they had consumed any product containing caffeine or ergogenic aid in the past 48 h. Participants were also requested to refrain from heavy exercise for 24 h and from food intake for 2 h prior to testing. Participants who were unable to complete the study within the three-week period were excluded from data analysis.

### Design and Procedures

This study was conducted using a double-blind, placebo-controlled, randomized design in order to determine the real and perceived effects of SS, caffeine and the combination of both. Participants had to visit the laboratory on seven separate occasions. During visit one, participants were familiarized with the WAnT standardized warm-up and the test protocol. During the remaining visits, participants completed all WAnT trials. These were performed on the same ergometer (Peak Bike 894, Monark, Vansbro, Sweden), where the saddles and handlebars were adjusted to suit each participant, and settings were replicated during each subsequent visit. The ergometer was calibrated before each test per the manufacturer's recommendations. To minimize training effects and prevent the effects of the circadian rhythm, each participant completed all seven visits within a period of 28 days and at the same time of the day (± 1 h). A minimum of the 72-h washout period separated visits that involved caffeine or placebo consumption from non-supplemented visits. Laboratory conditions were stable in a range of 22–23°C with 55–60% humidity. For the standardized warm-up (Demirarar and Cabuk, 2025), participants were asked to pedal for five minutes at a cadence of 70 revolutions per minute (RPM) against a 2-kg load. The warm-up during the 3^rd^, 4^th^, and 5^th^ min included three 5-s sprints where participants were instructed to cycle at their maximal cadence. Participants consequently completed a maximal 30-s WAnT under six conditions, namely a no-treatment control condition (WAnT_Con_), an SS condition (WAnT_SS_), a placebo (maltodextrin) condition (WAnT_P_), a placebo combined with SS condition (WAnT_PSS_), a caffeine condition (WAnT_C_) and a caffeine combined with SS condition (WAnT_CSS_). A deceptive protocol was used to examine any placebo effect and the effect of caffeine. Participants were informed about the involvement of a placebo condition but were unaware of having been given either the caffeine or the placebo. Neither the researchers nor the participants were aware of whether they received caffeine or the placebo. Only a single researcher, who was not involved in data collection, was aware of the condition assigned to each participant, ensuring the blinding principle of the study was maintained. The experimental design was a randomized, double-blind, placebo-controlled crossover study design. Participants were instructed to avoid intense exercise for 24 hours before the tests and to refrain from consuming any caffeine-containing beverages on the test day. Compliance with these restrictions was monitored through self-reported dietary logs and verbal confirmation before the test.

### Static Stretching Protocol

Before performing the WAnT-specific warm-up protocol, participants completed nine lower-body SS exercises. These were targeted at (1) gastrocnemius, (2) tibialis anterior, (3) hamstring muscles, (4) quadriceps muscle, (5) gluteus maximus, (6) iliopsoas, (7) hip adductor muscles, (8) hip abductor muscles, and (9) quadratus lumborum muscles. Stretches 1 and 4 to 9 were performed individually for the right and the left leg. Based on perceived stretch intensity, participants were required to hold each stretch for 30 s upon reaching their individual pain threshold. A 10-s passive rest interval was given between each SS exercise, and each stretch was performed twice (60 s of SS for each muscle/muscle group). The total duration of the SS protocol was ~22 min. Participants completed all stretches without assistance. The choice of SS exercise was related to the lower body muscles involved in cycling. After completion of the stretching exercises, the WAnT immediately commenced.

### Caffeine and Placebo Intake

Consumed caffeine reaches peak blood plasma levels after roughly 60 min (Pickering and Kiely, 2018). Therefore, participants were given the caffeine condition in a powder form with 300 ml of water or the placebo with 300 ml of water 60 min before the WAnT intervention. As recommended by the National Collegiate Athletic Association (NCAA), the amount of caffeine consumed was 6 mg∙kg^-1^. This concentration was replicated for the placebo (maltodextrin) condition. Caffeine or placebo intake was administered under the supervision of researchers upon the participants' arrival at the laboratory.

## Measures

### Familiarization Sessions

Since conducting familiarization sessions before the WAnT trial on the cycle ergometer leads to more valid and reliable results, all participants underwent familiarization sessions. Before starting these sessions, participants completed standardized warm-up protocols. The familiarization sessions consisted of two parts (Cabuk et al., 2025). In the first part, participants performed eight repetitions of 5-s maximal effort sprints against a resistance equal to 7.5% of their BM, with 40-s active recovery periods (unloaded pedaling at 50 RPM). Five minutes after completing the first part, participants proceeded to the second part of the familiarization session, where they performed a 30-s maximal effort sprint against a resistance corresponding to 7.5% of their body mass. During both exercise trials, the resistance was automatically applied when participants reached a pedaling cadence of 120 RPM, and the workload duration was initiated. This protocol ensured that all participants became accustomed to the test conditions before data collection. To minimize potential effects arising from postural changes, participants were instructed to maintain a seated position throughout the sessions.

### Wingate Anaerobic Test Protocol

The WAnT was based on 30-s maximal efforts against a load of 7.5% body mass (kg). Since the time to reach peak power should be less than 2 s, the initial (starting) cadence of the WAnT was fixed at 120 RPM. At the start of the test, participants were asked to accelerate. Upon reaching a pedal speed of 120 RPM, the load of the ergometer basket automatically dropped onto the flywheel, and a 30-s countdown started. Participants were encouraged throughout the test to exert maximum effort. They were also verbally informed about the elapsed time. After completing the 30-s effort, the weighted basket was lifted, and participants continued to cycle unloaded for a 5-min recovery period.

Five variables related to the evaluation of anaerobic performance were obtained from the WAnT: i) PPO, defined as the highest mechanical power achieved at any time during the 30-s test; ii) AvPO, considered as the average of the PO values obtained during the 30-s period; iii) POmin, defined as the lowest mechanical PO value; iv) maximal revolutions per minute (RPM_max_) considered the highest pedal revolution at any time period; and v) tPPO. Among these variables, PPO, RPM_max_, and tPPO were associated with anaerobic power, while AvPO and POmin were related to anaerobic capacity.

### Statistical Analysis

In the G*Power F-test a priori analysis conducted to determine the required sample size for the study, when the effect size (f) = 0.32, type I error rate (α) = 0.05, power (1-β) = 0.80, the number of groups = 1, and the number of measurements = 6 were selected, the required sample size was calculated as twelve. Therefore, twelve recreationally active males were recruited for the study. All data were assessed for normality using the Shapiro-Wilk test. The variables were analyzed using the one-way repeated measures ANOVA/LSD. Partial eta squared (η^2^_p_) values were calculated for ANOVA comparisons, with effect sizes classified as small (< 0.02), medium (0.02–0.26), or large (> 0.26). Additionally, effect sizes (ES) of the differences in pairwise comparisons were calculated according to the Cohen's *d* coefficient. The effect size of the differences was categorized as trivial (< 0.2), small (0.2–0.5), medium (0.5–0.8), and large effect (> 0.8). The statistical significance level was accepted at *p* < 0.05.

## Results

### Mechanical Power Output

PPO values varied significantly across conditions (F(5, 55) = 10, *p* = 0.000, η^2^_p_ = 0.477). The WAnT_Con_ condition produced significantly lower PPO (914 ± 109 W vs. 968 ± 108 W; *p* = 0.029) or higher PPO values (914 ± 109 W vs. 853 ± 103 W; *p* = 0.005) in comparison to the WAnT_C_ and the WAnT_SS_ conditions, respectively. The WAnT_SS_ condition resulted in significantly lower PPO values compared to the WAnT_CSS_ (853 ± 103 W vs. 932 ± 102 W; *p* = 0.004), WAnT_P_ (853 ± 103 W vs. 955 ± 110 W; *p* = 0.000), and WAnT_C_ conditions (853 ± 103 W vs. 968 ± 108 W; *p* = 0.000). The WAnT_SS_ condition resulted in statistically similar PPO value to the WAnT_PSS_ condition (853 ± 103 W vs. 886 ± 105 W; *p* = 0.118). Effect sizes are shown in Table 1.

**Table 1:** Statistical results of caffeine, static stretching, and placebo conditions on the Wingate test performance indices measured against the control, static stretching and combined with caffeine and static stretching condition.

		WAnT_Con_					WAnT_SS_					WAnT_CSS_				
		PPO	AvPO	PO_min_	tPPO	RPM_max_	PPO	AvPO	PO_min_	tPPO	RPM_max_	PPO	AvPO	PO_min_	tPPO	RPM_max_
WAnT_Con_	*p*						0.005	0.246	0.254	0.036	0.014	0.537	0.260	0.890	0.615	0.434
	ES						1.0	0.35	0.34	0.68	0.84	0.18	0.34	0.04	0.15	0.23
WAnT_SS_	*p*	0.005	0.246	0.254	0.036	0.014						0.004	0.025	0.078	0.760	0.000
	ES	1.0	0.35	0.34	0.68	0.84						1.05	0.74	0.56	0.09	1.49
WAnT_PSS_	*p*	0.352	0.702	0.328	0.158	0.353	0.118	0.136	0.403	0.873	0.296	0.001	0.083	0.525	0.447	0.007
	ES	0.28	0.11	0.29	0.43	0.28	0.49	0.46	0.25	0.04	0.31	1.39	0.55	0.19	0.22	0.95
WAnT_CSS_	*p*	0.537	0.260	0.890	0.615	0.434	0.004	0.025	0.078	0.760	0.000					
	ES	0.18	0.34	0.041	0.15	0.23	1.05	0.74	0.56	0.09	1.49					
WAnT_P_	*p*	0.070	0.524	0.329	0.158	0.094	0.000	0.085	0.654	0.025	0.000	0.084	0.660	0.198	0.396	0.190
	ES	0.58	0.19	0.29	0.43	0.53	1.90	0.54	0.13	0.74	2.41	0.54	0.13	0.39	0.25	0.40
WAnT_C_	*p*	0.029	0.008	0.702	0.982	0.018	0.000	0.001	0.270	0.223	0.000	0.066	0.058	0.332	0.631	0.042
	ES	0.72	0.93	0.11	0.008	0.80	1.63	1.35	0.33	0.37	2.52	0.58	0.60	0.29	0.14	0.66

Abbreviations: WAnT_Con_: a no-treatment control condition, WAnT_SS_: a static stretching condition, WAnT_PSS_: a placebo combined with static stretching condition, WAnT_CSS_: a caffeine combined with static stretching condition, WAnT_P_: a placebo condition, WAnT_C_: a caffeine condition, PPO: peak power, AvPO: average power, POmin: minimum power, tPPO: time to peak power, RPM_max_: maximal revolutions per minute, p: level of significance, and ES: effect size

AvPO values significantly differed across the conditions (F(5, 55) = 4.41, *p* = 0.002, η^2^_p_ = 0.286). The WAnT_SS_ condition resulted in significantly lower AvPO values compared to the WAnT_CSS_ (566 ± 70 W vs. 578 ± 74 W; *p* = 0.025) and WAnT_C_ conditions (566 ± 70 W vs. 586 ± 72 W; *p* = 0.001)_._ The AvPO under the WAnT_C_ condition was higher than under the WAnT_Con_ (586 ± 72 W vs. 573 ± 74; *p* = 0.008), WAnT_SS_ (586 ± 72 W vs. 566 ± 70; *p* = 0.001), WAnT_PSS_ (586 ± 72 W vs. 571 ± 72; *p* = 0.010), and WAnT_P_ conditions (586 ± 72 W vs. 577 ± 68; *p* = 0.042), except for the WAnT_CSS_ condition (586 ± 72 W vs. 578 ± 74; *p* = 0.058).

POmin values did not differ significantly across the conditions (F(5, 55) = 0.78, *p* = 0.563, η^2^_p_ = 0.067).

#### Time to Reach Peak Power and Maximal Revolutions per Minute

Although the one-way repeated measures ANOVA did not reveal significant differences in tPPO across conditions (F(1.84, 20.2) = 1.15, *p* = 0.333, η^2^_p_ = 0.095), pairwise comparisons indicated that tPPO was significantly longer under the WAnT_SS_ condition compared to WAnT_Con_ (1.59 ± 0.7 s vs. 1.26 ± 0.4 s; *p* = 0.006) and WAnT_P_ conditions (1.59 ± 0.7 s vs. 1.12 ± 0.1 s; *p* = 0.038).

**Figure 1:**
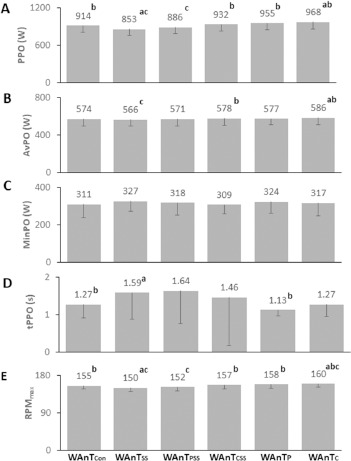
Effects of caffeine, static stretching, and placebo conditions on the Wingate test performance indices measured against the control and the static stretching condition. Abbreviations: WAnT_Con_: a no-treatment control condition , WAnT_SS_: a static stretching condition, WAnT_PSS_: a placebo combined with static stretching condition, WAnT_CSS_: a caffeine combined with static stretching condition, WAnT_P_: a placebo condition, WAnT_C_: a caffeine condition, PPO: peak power, AvPO: average power, POmin: minimum power, tPPO: time to peak power, RPM_max_: maximal revolutions per minute; ^a^ significantly different compared to WAnT_Con_ , ^b^ significantly different compared to WAnT_SS_, ^c^ significantly different compared to WAnT_CSS_

Significant differences in RPM_max_ values were observed among the conditions (F(2.05, 22.5) = 10.4, *p* = 0.001, η^2^_p_ = 0.49). The WAnT_Con_ condition reached significantly higher RPM_max_ and lower RPM_max_ values compared to the WAnT_SS_ (155 ± 7.21 RPM vs. 150 ± 8.34 RPM; *p* = 0.014) and the WAnT_C_ (155 ± 7.21 RPM vs. 160 ± 8.6 RPM; *p* = 0.000) conditions. Except for the WAnT_PSS_ condition (152 ± 10.3 RPM vs. 150 ± 8.34 RPM; *p* = 0.296), the WAnT_SS_ condition resulted in significantly lower RPM_max_ values (*p* = 0.000–0.014) than all other conditions (Figure 1 and Table 1). However, the WAnT_CSS_ condition resulted in significantly higher RPM_max_ values compared to WAnT_SS_ (157 ± 10 RPM vs. 150 ± 8.34 RPM; *p* = 0.000) and WAnT_PSS_ conditions (157 ± 10 RPM vs. 152 ± 10.3 RPM; *p* = 0.007) and in significantly lower RPM_max_ value compared to the WAnT_C_ condition (157 ± 10 RPM vs. 160 ± 8.6 RPM; *p* = 0.042) (Figure 1 and Table 1).

## Discussion

Our main findings reveal that the WAnT_SS_ condition led to significant performance impairment in PPO, RPM_max_, and tPPO values compared to the WAnT_Con_ condition (Figure 1). On the other hand, when participants ingested caffeine in addition to SS (WAnT_CSS_), they demonstrated significantly higher PPO, AvPO, and RPM_max_ values compared to the conditions involving SS (WAnT_SS_ and WAnT_PSS_) (Figure 1). These results support our first hypothesis that SS reduces anaerobic performance and our second hypothesis that combining SS with caffeine intake can mitigate this reduction. Additionally, significant differences in PPO, AvPO, and RPM_max_ values were observed between the WAnT_Con_ and WAnT_C_ conditions, with effect sizes ranging from 0.72 to 0.93 (Figure 1 and Table 1). However, when comparing WAnT_CSS_ to WAnT_Con_ condition, statistically similar results were found for these variables, with smaller effect sizes ranging from 0.18 to 0.34, suggesting that SS may limit the ergogenic effects of caffeine (Figure 1 and Table 1).

In relation to our first hypothesis, our results are consistent with some studies in the literature examining the effects of SS on anaerobic performance (Ramirez et al., 2007; Velasque et al., 2020), while they differ from others. Velasque et al. (2020) observed decreases of 6.9% and 4.2% in PPO and AvPO, respectively, in less fit soccer players performing the WAnT following an SS protocol (2 × 2 SS exercises × 45 s, r: 60 s) compared to a traditional cycling warm-up. Similarly, Ramirez et al. (2007) compared the results of a 30-s WAnT performance after an SS protocol to a traditional cycling warm-up protocol and found that PPO was 15.3% lower and AvPO was 5.9% lower after SS. In contrast, the results of Franco et al. (2012) and Miller (2020) contradict the results of our study. Those studies did not observe significant differences in AvPO and PPO values. However, they reported decreases in POmin and prolonged tPPO duration following SS compared to a traditional cycling warm-up (3 × 3 SS exercises × 30 s) (Franco et al., 2012). Similarly, Miller (2020) found a decrease in PPO values following an SS protocol (4 × 4 SS exercises × 30 s) in the WanT, but did not report significant differences in AvPO and POmin values.

Factors such as the duration of each repetition in SS protocols, total stretching duration, the type of SS, and the muscle groups targeted during stretching exercises may explain our results and the conflicting findings in the literature (Chaabene et al., 2019; Franco et al., 2008; Oshita et al., 2016). In the present study, the WAnT_SS_ compared to the WanT_Con_, showed reductions in PPO, and RPM_max_ values, which are associated with anaerobic power, while POmin and AvPO values remained similar. Our study is supported by current research results regarding individual responses to SS and caffeine intake (Table 2). Overall, our study results confirm the negative effects of SS on anaerobic performance while suggesting that caffeine intake may counteract these effects. To understand the underlying mechanisms of these differences, examining the neuromuscular system’s response is essential. A reduction in neuromuscular input may lead to decreased muscle force production and contraction speed, explaining the detrimental effects of SS on performance. Various studies have shown that prolonged SS applications result in reductions in anaerobic performance and neuromuscular input (Behm et al., 2016; Chaabene et al., 2019; Konrad et al., 2019). Behm et al. (2016) suggested that the most likely reason for performance impairment following SS was a decrease in CNS excitation. Similarly, Trajano et al. (2014) emphasized that the decline in maximal strength performance after SS was solely due to neural factors. Several studies have observed decreased electromyographic (EMG) activity in the muscles involved following prolonged SS (Marek et al., 2005; Trajano et al., 2013). For example, SS protocols that hold the muscle under tension for more than 60 s have been reported to reduce the neural drive that the muscle can generate during maximal voluntary contraction (i.e., lower motor unit activation) (Palmer et al., 2019). Researchers such as Hough et al. (2009) and Trajano et al. (2013) reported that motor unit activation directed to the muscles decreased after SS, meaning the muscle was unable to recruit as many motor units as before to produce the same level of force. One possible explanation for this is the decreased sensitivity of muscle spindles during SS and the suppression of reflex activity at the spinal level (Behm et al., 2016). As a result, the reduced excitability or inhibition of α-motor neurons may decrease motor unit activity. The performance impairment observed in the WAnT following SS can be explained by these mechanisms.

**Table 2:** Individual subject data.

		1	2	3	4	5	6	7	8	9	10	11	12
PPO (W)	WAnT_Con_	936	860	1066	1056	983	753	784	1068	882	802	886	896
	WAnT_SS_	920	730	953	1049	913	707	793	915	731	811	846	868
	WAnT_PSS_	936	811	927	997	972	736	849	994	680	1004	881	851
	WAnT_CSS_	1001	833	980	982	985	809	865	1093	740	1038	928	933
	WAnT_P_	980	841	1086	1069	1015	791	848	1094	803	1027	955	955
	WAnT_C_	1045	868	1111	1008	1065	808	803	1066	854	1013	1032	947
AvPO (W)	WAnT_Con_	611	511	755	629	543	480	502	599	605	548	530	571
	WAnT_SS_	613	488	734	618	549	481	514	595	546	541	523	595
	WAnT_PSS_	608	504	743	620	550	468	519	622	546	554	531	590
	WAnT_CSS_	602	514	769	622	544	477	526	618	583	561	534	592
	WAnT_P_	601	506	734	621	541	471	527	618	601	556	548	597
	WAnT_C_	627	525	764	627	567	481	520	603	597	559	552	613
POmin (W)	WAnT_Con_	325	321	400	350	217	263	277	412	409	212	264	278
	WAnT_SS_	329	299	444	348	282	233	299	376	375	338	282	326
	WAnT_PSS_	348	280	436	336	268	265	297	398	403	231	270	284
	WAnT_CSS_	255	270	357	337	242	249	297	362	397	323	288	326
	WAnT_P_	346	313	447	305	229	232	301	384	383	330	279	344
	WAnT_C_	231	288	436	349	257	231	291	372	423	324	274	321
tPPO (s)	WAnT_Con_	1.99	1.01	2.00	1.01	1.02	1.27	1.31	1.03	1.35	1.02	1.16	1.05
	WAnT_SS_	1.94	1.56	3.53	1.03	1.04	2.17	1.46	1.05	1.45	1.09	1.49	1.28
	WAnT_PSS_	1.11	1.02	2.82	1.8	1.05	1.02	1.26	1.57	3.76	1.05	1.05	2.12
	WAnT_CSS_	1.08	1.08	1.49	1.04	1.03	1.09	1.03	1.13	5.51	1.02	1.02	1.00
	WAnT_P_	1.08	1.12	1.53	1.00	1.05	1.01	1.11	1.08	1.05	1.14	1.02	1.34
	WAnT_C_	1.13	1.02	1.08	2.19	1.11	1.3	1.52	1.14	1.31	1.11	1.04	1.31
RPM_max_	WAnT_Con_	150	157	142	168	161	152	147	164	154	151	157	153
	WAnT_SS_	153	148	134	166	155	145	147	153	138	156	153	149
	WAnT_PSS_	153	154	133	156	157	150	153	158	138	173	155	145
	WAnT_CSS_	158	156	138	168	159	157	156	168	138	168	159	157
	WAnT_P_	157	156	142	171	161	154	155	168	147	170	163	154
	WAnT_C_	163	159	143	169	164	156	151	168	151	171	166	156

Abbreviations: WAnT_Con_: a no-treatment control condition, WAnT_SS_: a static stretching condition, WAnT_PSS_: a placebo combined with static stretching condition, WAnT_CSS_: a caffeine combined with static stretching condition, WAnT_P_: a placebo condition, WAnT_C_: a caffeine condition, PPO: peak power, AvPO: average power, POmin: minimum power, tPPO: time to peak power, RPM_max_: maximal revolutions per minute

The ability of caffeine intake to counteract WAnT performance declines caused by SS can be explained by its role in the CNS. Caffeine acts as an antagonist at adenosine receptors, blocking the inhibitory effects of adenosine in the CNS. As a result, neuronal activity increases, leading to enhanced alertness and concentration. The blockade of adenosine results in increased neurotransmitter release, such as dopamine and norepinephrine, which facilitates voluntary muscle activation by increasing motor unit firing rates and sustaining neuro-excitability (Davis and Green, 2009; de Kivit et al., 2013; Fredholm et al., 1999). Consequently, it is expected that caffeine can reduce neuromuscular inhibition caused by SS. Indeed, it has been proposed that the performance loss observed after SS is due to increased neuromuscular inhibition originating from the CNS and that this inhibitory effect can be mitigated by caffeine, a known stimulant (Behm et al., 2016). Although our study did not measure these physiological responses, the fact that the WAnT_CSS_ condition counterbalanced the reductions in PPO, AvPO, and RPM_max_ caused by SS suggests that this effect may be attributed to caffeine’s ability to block adenosine receptors in the CNS.

To the best of our knowledge, only one study in the literature (Farney et al., 2019) has examined whether caffeine intake can prevent performance declines caused by SS. In that study, the authors investigated the effects of a 6 mg∙kg^-1^ caffeine dose and an SS protocol consisting of 4 × 4 SS exercises × 30 s on the 1RM knee flexion test results. Following caffeine or placebo intake, participants experienced an ~7% decrease in 1RM knee flexion performance after the SS protocol. However, it was determined that caffeine intake did not significantly reduce these SS-induced performance declines. Furthermore, the similarity of the 1RM knee flexion test results obtained under caffeine and placebo conditions indicates that participants did not exhibit an ergogenic response to caffeine. Therefore, the absence of any observable effect on performance losses induced by SS can be considered an expected outcome. However, the lack of a non-intervention control condition and an independent SS protocol in that study may have been insufficient to fully determine the effects of both caffeine and SS-induced ergogenic responses. The review study by Davis and Green (2009) and the systematic review and meta-analysis by Polito et al. (2016) have concluded that caffeine intake enhances muscular endurance performance but has no effect on maximal strength. Similarly, Wilk et al. (2019) noted that the effects of caffeine on maximal strength performance remained inconclusive. While Farney et al. (2019) found that caffeine intake had no effect on performance loss caused by SS, our study demonstrated that this loss was counteracted. This difference may be due to the varying effects of caffeine on performance test variables or methodological differences in the applied protocols.

In addition to the variation in caffeine’s effects depending on the performance test variable, studies reporting individual participant data have revealed significant inter-individual variability in response to caffeine intake (Grgic and Mikulic, 2017; Pickering and Kiely, 2018). For example, Grgic and Mikulic (2017) investigated the effects of caffeine and placebo intake on the 1RM back squat test in 17 participants. Of these, 11 participants demonstrated higher strength values after caffeine intake, while three performed better under the placebo condition, and in three participants, no difference was observed between the caffeine and the placebo condition. These differences may be influenced by various factors, including individual characteristics, training status, genetic variations, caffeine sensitivity, the type and the dose of caffeine intake, time to peak caffeine concentration in the blood, and the method used to assess anaerobic performance (e.g., sprint, vertical jump) (Davis and Green, 2009; Grgic, 2018; Grgic and Mikulic, 2017; Pickering and Kiely, 2018). Moreover, the effects of caffeine intake can also be influenced by both positive and negative ergogenic expectancy effects (Beedie et al., 2007). Although performance responses to caffeine may be influenced by the factors mentioned above, the International Olympic Committee’s (2018) consensus statement on dietary supplements recognizes caffeine as one of the few supplements with well-established ergogenic effects on performance (Maughan et al., 2018).

In our study, the WAnT_C_ condition led to significantly increased PPO, AvPO, and RPM_max_ values by 5.6%, 2.2%, and 3.1%, respectively, compared to the WAnT_Con_ condition. These results align with the results of a comprehensive meta-analysis conducted by Grgic (2018), which demonstrated that caffeine intake increased PPO and AvPO values in the WAnT by 3% and 4%, respectively (Grgic, 2018). Notably, in our study, the WAnT_C_ condition showed a marginally significant advantage over the WAnT_P_ condition in terms of RPM_max_ (*p* = 0.049) and AvPO (*p* = 0.042), while PPO, POmin, and tPPO values remained similar. Given that all participants were aware they had consumed caffeine and considering the widespread belief in caffeine’s ergogenic effects, the performance improvement observed under the placebo condition may be attributed to this expectancy effect. However, the observed improvements in PPO, AvPO, and RPM_max_ under the WAnT_CSS_ compared to the WAnT_SS_ condition, while no such improvements were found under the WAnT_PSS_ condition, provide strong evidence that caffeine enhances performance through pharmacological mechanisms rather than expectancy alone. If caffeine were solely an ergogenic aid through expectancy, the WAnT_CSS_ condition would not have counteracted the performance decline caused by WAnT_SS_ when compared to WAnT_Con_. These results emphasize that the placebo or expectancy effect is less effective in enhancing performance compared to caffeine’s pharmacological effects.

On the other hand, Beedie et al. (2006) observed that participants who believed they had consumed caffeine but were actually given a placebo reported typical caffeine-related symptoms such as increased energy and concentration. Their study suggested that placebo-induced arousal could mimic the real effects of caffeine. If this were the case in our study, the placebo and SS combination would have been expected to counteract the performance impairment caused by SS. However, our results indicate that the placebo condition’s effects on performance were limited to psychological expectations, whereas caffeine enhanced performance through both psychological expectations and pharmacological mechanisms. These results demonstrate that caffeine intake before SS effectively counteracts the negative effects of SS and confirms the practical and real ergogenic effects of caffeine.

This study has some limitations that should be acknowledged. In our study, no assessment of caffeine intolerance was conducted among the participants. Only one participant exhibited symptoms of nausea following caffeine intake and was therefore excluded from the study. However, no adverse effects related to caffeine consumption were observed in the other participants. The relatively small sample size and the inclusion of only male participants may limit the generalizability of the results. The inclusion of only male participants was intended to reduce potential variability and to obtain a more homogeneous sample. The presence of conflicting findings in the literature regarding sex-specific responses to caffeine intake was also a factor influencing this decision. While some studies have reported significant sex-related differences, others have found no meaningful differences. However, the exclusion of female participants limits the generalizability of the findings.

## Conclusions

The WAnT results show a high degree of consistency with other laboratory and field tests designed to assess anaerobic performance (Del Coso and Mora-Rodriguez, 2006). Therefore, the 2–8.5% improvements in PPO, AvPO, and RPM_max_ variables observed with the combination of SS and caffeine, compared to SS alone, may be practically significant for sports that align with WAnT characteristics (e.g., sprinting, the long jump, the high jump, the shot put, the javelin throw, etc.). For elite athletes, even a 1% decrease in performance can result in failing to qualify for the Olympics, a change in medal color, or missing out on a medal entirely. For instance, in the men’s 100-m final at the 2024 Paris Olympics, the finishing times of the gold (9.79 s), silver (9.79 s), and bronze (9.81 s) medalists, as well as the fourth-place finisher (9.82 s), were separated by just three hundredths of a second. This highlights how even a 1% change in performance can be critical. Such small differences are also crucial in team sports where anaerobic performance plays a decisive role. For example, Cin et al. (2021) emphasized that, given the characteristics of volleyball, even a 1-cm difference in vertical jump height can provide a significant advantage when blocking at the net.

The results of this study confirm the negative effects of SS on anaerobic performance while demonstrating that caffeine intake may minimize or counterbalance these effects. Our results suggest that consuming 6 mg∙kg^-1^ of caffeine 60 min before the SS protocol may serve as a potential strategy to prevent anaerobic performance loss. Given that even a 1% increase in anaerobic performance can provide a significant competitive advantage for athletes, our results offer a practical approach for pre-performance applications. Since SS is also commonly used by recreational athletes, our results are relevant not only for competitive athletes but also for recreational sports participants. These results have implications for a broad range of athletes involved in sports requiring anaerobic power and anaerobic capacity. Additionally, the potential risk that SS may partially diminish the positive effects of caffeine should not be overlooked. Given that individual variability can influence these responses, appropriate pre-competition trials should be conducted to determine optimal strategies.

Future studies with larger sample sizes, the inclusion of female athletes, and participants with different training backgrounds could provide more comprehensive and reliable insights into the effects of SS and caffeine intake on anaerobic performance. Although the WAnT test shows a high degree of consistency with both laboratory and field tests, further studies examining the reproducibility of these results using anaerobic power and capacity-related field tests are necessary. Additionally, since different participant profiles and test variables can influence the effects of caffeine intake and SS on performance, further research is required to determine whether caffeine intake can consistently counteract performance impairment caused by SS. Finally, investigating the interactive effects of caffeine intake with different types of stretching, such as dynamic, ballistic, and proprioceptive neuromuscular facilitation, or with combined stretching protocols may provide further insights into optimal warm-up strategies for enhancing anaerobic performance.
